# Differences in Strength, Muscle Work, and Hamstring/Quadriceps Ratio in Professional and Junior Elite Basketball Players According to Sex

**DOI:** 10.3390/jfmk10020204

**Published:** 2025-06-02

**Authors:** Raúl Coto Martín, Néstor Pérez Mallada, María Jesús Martínez Beltrán, Lucía Cuéllar Marín, Pablo José Borrás Luján, Oscar Otín Arroyo, María Ana Sáenz-Nuño, Juan Manuel Arribas-Marín

**Affiliations:** 1 San Juan de Dios Foundation, 28016 Madrid, Spain; rcoto@comillas.edu (R.C.M.); mjesus.martinez@comillas.edu (M.J.M.B.); lcuellar@comillas.edu (L.C.M.); juanmarribas@comillas.edu (J.M.A.-M.); 2 Health Sciences Department, San Juan de Dios School of Nursing and Physical Therapy, Comillas Pontifical University, 28350 Madrid, Spain; 3 Institute for Research in Technology (IIT), ICAI School of Engineering, Comillas Pontifical University, 28015 Madrid, Spain; msaenz@iit.comillas.edu; 4 GICAF Research Group, Department of Education, Research Methods and Evaluation, Faculty of Human and Social Sciences, Universidad Pontificia Comillas, 28049 Madrid, Spain; 5 Club Estudiantes de Baloncesto, 28006 Madrid, Spain; pborras@clubestudiantes.com (P.J.B.L.); o.otin@clubestudiantes.com (O.O.A.)

**Keywords:** anterior cruciate ligament (ACL) injury, strength ratio, isokinetic strength, hamstrings, quadriceps, basketball players

## Abstract

**Background:** Anterior cruciate ligament (ACL) injury is more prevalent in young athletes and women. A deficit of knee flexor strength and a H/Q strength ratio below 0.6 in athletes are risk factors. Therefore, the main objective of this study is to determine if there are differences in the strength of the knee musculature, joint work, and H/Q ratio between professional and junior elite basketball players and sexes, since there are differences in the incidence of ACL injuries between these groups. **Methods**: Forty-three professional (27 male and 16 female) and 42 junior elite (28 male and 14 female) basketball players were evaluated. Dynamometric knee flexion and extension measurements were performed on both lower limbs at three angular velocities: 30°/s, 120°/s, and 180°/s. Concentric knee flexion and extension strength variables were measured in Newtons, the strength ratio between flexors and extensors in H/Q ratio, and muscle work (product of force times displacement) in Joules. Comparisons of the measured variables were made between categories and sexes. **Results**: Significant differences (*p* < 0.05) were found between categories (professional and junior) in all measured strength parameters as well as in joint work. No differences were found between categories in the H/Q ratios. Significant differences (*p* < 0.05) were found between sexes in most measured strengths and joint work. However, no differences were found in most of the H/Q ratios. Female basketball players have lower values of strength and muscle work than male players, however, in the H/Q ratio data there are no differences between the two groups. The same occurs between junior and professional athletes. **Conclusions**: The authors conclude that flexor and extensor strength values should be analyzed in isolation and not in terms of the H/Q ratio.

## 1. Introduction

Anterior cruciate ligament (ACL) injury is one of the most common and serious knee injuries in sports [1,2,3,4] and is increasing annually by 1.3% [5]. There is a higher incidence of this injury in sports such as basketball, with continuous changes of direction, jumps, and landings [4]. Notably, ACL injury occurs more frequently in female athletes between the ages of 15 and 19 years and male athletes between the ages of 20 and 24 years [6]. Previous studies have reported a higher incidence of ACL injuries in female athletes (3.5%) compared to male athletes (2%) [7]. These injuries are three to six times more common in female athletes [3], and the risk may be up to 10 times higher among females participating in pivoting sports such as basketball and soccer [8].

However, it is important to note that the prevalence of ACL injuries may vary depending on the athlete’s age. Benis et al. [9] evaluated the incidence of ACL injuries in elite basketball players aged 11 to 30 years. The study reported that 13.8% of injuries occurred in athletes aged 11–15 (Tier 2, intermediate-level athletes [10]), 52.5% in athletes aged 16–20 (Tier 3, advanced-level athletes [10]), and 16.9% in athletes aged 21–25 and 26–30 (Tier 4, elite-level athletes [10]). These results highlight the significant differences in injury prevalence across age groups and performance levels [9]. The greatest risk of injury in the young population may be linked to the body maturation process. It is important to note that the rapid growth of bones during puberty does not match the speed of muscle growth, which is slower. This can lead to a loss of flexibility, which in turn is associated with a higher risk of injury. Additionally, in women, the increase in strength of the knee flexor-extensor muscles after the peak growth period is not only smaller but also delayed compared to men. Similarly, the increase in hamstring muscle strength in women is less than that of the quadriceps muscles [11]. In terms of hormonal factors, women who have gone through menarche are up to 6.7 times more likely to suffer an ACL injury compared to prepubescent athletes who have not yet menstruated. In relation to this, when a woman is in the pre-ovulatory phase of the menstrual cycle, she has two to three times more risk of injury compared to other stages of the cycle. High concentrations of estrogens, such as estradiol, can negatively affect the ligament structure and, consequently, its mechanical function [12].

Biomechanical alterations in movement patterns may also favor the risk of suffering this injury. Alterations such as dynamic knee valgus [1,2], less pelvic anteversion [13], a deficit of ankle dorsal flexion [14], less ground contact time in landing after jumping [15], and a decrease in hamstring strength reduces the ability to control the tibia and increasing its anterior translation, which causes the ligament to bear more load. Additionally, when the quadriceps are stronger than the hamstrings, this muscle group will also contribute to the anterior translation [1].

ACL tears are commonly associated with an imbalance between the knee quadriceps extensor and hamstring flexor musculature (H/Q ratio) [16,17], as well as a decrease in hamstring strength [2,18,19]. The cut-off point for this ratio is set at 0.6; below this, the risk of associated hamstring musculature or ACL injuries increases [16]. However, there is evidence that it is impossible to predict ACL injuries with conventional H/Q ratio data since it does not consider the angle where the peak of maximal force occurs [20]. Regarding joint work (the product of force and displacement), there is little literature on the subject, but we believe it is a variable that provides us with interesting data on the athlete’s functionality [21]. Several recent studies have highlighted that a low H/Q ratio, particularly under dynamic conditions, may contribute to the risk of ACL re-injury. However, they also emphasize that its predictive value is limited when considered in isolation and should be interpreted alongside other neuromuscular or biomechanical variables [22,23]. The inclusion of joint work aims to capture not only the peak strength but also functional capacity during movement, providing a more comprehensive assessment of knee musculature performance under load. This integrated approach may more accurately reflect real sport conditions and improve the sensitivity in identifying athletes at risk.

The primary objective of this study is to investigate whether significant differences exist in knee flexion and extension strength, joint work, and the H/Q ratio among male and female professional and junior basketball players. Given that ACL injuries are frequently associated with muscular imbalances and alterations in the H/Q ratio, this study seeks to shed light on how these variables may vary by gender and level of experience. The central hypothesis posits that female and junior athletes would present lower values in knee flexor and extensor strength, H/Q ratio, and joint work, factors that may contribute to their elevated risk of ACL injuries. This hypothesis is informed by the existing literature, demonstrating that reduced joint work capacity and compromised H/Q ratios heighten susceptibility to knee injuries. By focusing on this biomechanical interplay, the study aims to contribute nuanced insight into injury risk profiling and inform targeted, evidence-based prevention strategies.

## 2. Materials and Methods

### 2.1. Experimental Design

A cross-sectional study was carried out, with data collected from clinical reports of the biomechanical tests of basketball players used for injury prevention in 2021–2022. All reports belonging to players with sports medical discharge were included. Reports belonging to players on medical leave at the time of measurement or who had previously suffered an ACL injury were excluded.

Informed consent for data analysis was obtained for research purposes before the measurements. The study is based on the ethical principles for medical research involving human beings as declared in Helsinki in 1964 and the latest update in Brazil in 2013. Additionally, regarding the use of databases, the principles of the Taipei Declaration were considered.

The study was approved by the Clinical Research Ethics Committee of the Hospital Clínico San Carlos of Madrid, Spain, with approval number C.P.-C.I. 23/704-E, 4 December 2023.

Isokinetic strength measurement tests were performed in the biomechanics laboratory of the San Juan de Dios School of Nursing and Physiotherapy, Comillas Pontifical University.

### 2.2. Subjects

Eighty-five highly competitive basketball players were evaluated. Forty-three were professionals (between 19 and 32 years old) with a mean mass, height, and BMI of 85.7 ± 14.4 kg, 1.92 ± 0.14 m, and 23.01 ± 1.51, respectively (27 males with a mean mass of 93.67 ± 9.90 kg, height of 2.00 ± 0.09 m, and BMI 23.37 ± 1.19 and 16 females with a mean mass of 72.25 ± 10.20 kg, height of 1.79 ± 0.09 m, and BMI 22.4 ± 1.83).

A sample size calculation was performed by comparing the means to a reference value. A statistical power of 80% (1-beta = 0.80) and a significance level of 5% (alpha = 0.05) were applied. To determine the standard deviation and the minimum detectable difference (10% of the mean), the peak knee flexion strength in basketball players (88.8 ± 18.6) reported by Kyung Kin et al. [24] was used. An additional 10% was included to account for potential dropouts, resulting in a final estimated sample size of *n* = 39. Upon arrival at the laboratory, mass and height data were collected, from which the BMI was calculated.

### 2.3. Procedure

The PRIMUS RS 701 dynamometer (BTE Technologies, Hanover, MD, USA) was used to perform the concentric strength tests, as this type of device is considered the gold standard for measuring muscular strength [21,25]. The participants were placed in a seated position with the hip flexed at 110°. No support straps were placed on the trunk, hip, or thigh to avoid generating more fixed supporting points than the pad itself. The axis of the dynamometer was correctly aligned with the axis of motion of the knee joint. The pad was positioned with a 30 cm lever arm on all subjects. Measurements were performed at three different angular velocities: at 30°/s, three repetitions of flexion–extension were performed; at 120°/s, five repetitions were performed; and at 180°/s, ten repetitions were performed [26]. Before taking the measurements, a warm-up of ten submaximal flexo-extensions of the knee at 180°/s was performed to familiarize the participants with the device. A 2 min rest was given between each measurement.

The H/Q ratio was calculated by dividing the hamstring musculature’s maximum peak strength but the quadriceps musculature’s maximum peak strength at each measurement.

The joint work was automatically calculated by the Primus isokinetic device as the product of the measured force and the distance covered during the movement from full extension to 90° of knee flexion, in accordance with the range established by the testing protocol and considering the length of the applied lever arm. The total work for each angular velocity corresponds to the sum of the work performed across all repetitions executed at that speed.

### 2.4. Statistical Analysis

Statistical analysis was performed using the IBM SPSS^®^ software (version 23.0, Armonk, NY, USA) to determine whether significant differences exist between the categories (professional and junior) and/or sex. The Kolmogorov–Smirnov test was used to determine the normal or non-normal distribution of the variables. The nonparametric Mann–Whitney U test was used for variables that did not follow a normal distribution, and the Student’s *t*-test was used for independent samples if they did follow a normal distribution. For the analysis of the differences between sexes in each of the categories, the non-parametric Mann–Whitney U test was used in all statistical analyses because the sample size in these cases is *n* < 30. A comparison was performed on each leg for each angular velocity using the peak knee flexion strength, peak knee extension strength, work, and H/Q ratio variables as well as mass, height, and BMI.

A significance level of *p* < 0.05 was set for all statistical analyses *(p* < 0.05 was considered significant). Effect sizes were calculated using Cohen’s *d* for parametric tests (e.g., Student’s *t*-test), and *r* for non-parametric tests (e.g., Mann–Whitney *U* test), depending on the normality of the data. An effect size of 0.2 was considered small, 0.5 medium, and 0.8 or greater large.

## 3. Results

Significant differences were found between male and female professional players in mass and height (*p* ≤ 0.001) but not in BMI (*p* = 0.059). The remaining 42 subjects were junior athletes (between 16 and 18 years old) with a mean mass, height, and BMI of 65.17 ± 9.40 kg, 1.75 ± 0.07 m, and 21.28 ± 2.63, respectively (28 males with mean mass 66.00 ± 9.28 kg, height 1.78 ± 0.06 m, and BMI 20.76 ± 2.46 and 14 females with mean mass 63.46 ± 9.93 kg, height 1.70 ± 0.07 m, and BMI 22.34 ± 2.73). Significant differences were found between male and female junior players in height (*p* ≤ 0.001) but not in mass (*p* = 0.268) or BMI (*p* = 0.081). Significant differences in mass, height, and BMI (*p* ≤ 0.001) were found between professional and junior athletes. In addition, according to the sport level classification described by McKay et al. [10], professional players are considered Tier 4 athletes and junior players are considered Tier 3 athletes. More details about the subjects included in our study can be found in Table 1 and Table 2.

Statistically significant differences were found between professional and junior athletes in all flexion and extension strength variables measured. Additionally, muscle work in professional athletes at all angular velocities measured was greater in all cases (*p* < 0.05 and *d* > 1). However, no significant differences in H/Q ratios (*p* > 0.05) were found between the two groups (Table 3).

### 3.1. Sex Differences in Professional Basketball Players

The differences in the variables measured between the sexes (male and female) were evaluated. In the case of professional athletes (Table 4), significant differences were found between sexes in all flexion and extension strength variables measured, at all measured velocities, being higher in all cases in male athletes (*p* < 0.05), except for extension strength in the right knee at 120°/s and in the left knee at 30°/s. However, the effect size was high in both cases (0.78 and 0.64, respectively). Thus, there is a tendency at the clinical level to have a significant difference, since more than 78% in the first case and 74% in the second of the values obtained in the male category were above the mean of the value of the extension strength at these velocities of the category of female professional athletes.

In all muscle work values, differences were found between sexes in both knees at all measured velocities. In all cases, the values were higher in males.

As for the H/Q ratios, significant differences (*p* < 0.05) were found in the right knee at 180°/s and in the left knee at 30°/s and at 180°/s in males.

### 3.2. Sex Differences in Elite Junior Basketball Players

In the case of the junior category (Table 5), significant differences were observed between male and female players in many of the evaluated variables. In the right knee, significant differences (*p* < 0.005) between sexes were found in the extension strength at 30°/s and 120°/s, being greater in males in both cases. In the left knee, significant differences (*p* < 0.005) between sexes were found in the flexion strength at 120°/s and the extension strength at 120°/s, which were higher in males. Although no significant differences were found in the rest of the strength variables measured, the effect size could be considered high. In the right knee, both the flexion strength at 30°/s and 120°/s and the extension strength at 180°/s had an effect size between 0.6 and 0.7; thus, between 73% and 76% of the values obtained in the male category were above the mean value of the strength at these velocities of the female group. In the case of the left knee, the effect size was even larger in the flexion strength at 30°/s and 180°/s and extension at 30°/s and 180°/s (0.77, 0.77, 0.66 and 0.89, respectively).

In the case of muscular work, significant differences (*p* < 0.05) were detected at all measured velocities and in both knees of males.

As for the H/Q ratios, no significant differences were found between men and women in any of the ratios measured.

## 4. Discussion

In this study, professional athletes reported significantly higher isokinetic knee flexion strength compared to junior athletes. However, no differences were observed in the H/Q ratios. This difference in muscle strength may be a key factor underlying the higher incidence of ACL injuries reported in junior athletes [12], who demonstrate notably lower strength levels despite facing comparable athletic demands.

Our results are consistent with previous studies suggesting that differences in muscle strength may be a crucial factor. For instance, Ishøi et al. [27] reported that professional soccer players (Tier 4 athletes [10]) developed 16% less isometric knee flexion strength than elite U19 players (Tier 3 athletes [10]). However, our findings do not fully align with these results, as we observed that junior athletes had lower knee flexion and extension strength compared to professional athletes.

In a study by Nagai et al. [28], knee flexor and extensor strength were evaluated at 240°/s in high school basketball players. Among male athletes, quadriceps strength averaged 163 ± 23 N at age 16, declined by 14% at age 17, and then recovered by age 18. Similarly, hamstring strength decreased by 12% at age 17 compared to age 16 (78 ± 16 N), followed by a 23% increase at age 18 relative to age 17. This pattern of strength development may help explain the higher incidence of ACL injuries at younger ages. In our study, knee flexor and extensor strength were also found to be more developed in professional athletes compared to junior athletes, supporting these findings.

The scoping review by Van Melick et al. [26] supports our findings, concluding that elite junior athletes (Tier 3 [10]) have lower knee flexor and extensor strength than professional athletes (Tier 4 [10]) at both 60°/s (97 ± 18 Nm vs. 138 ± 4 Nm and 182 ± 28 Nm vs. 239 ± 16 Nm, respectively) and 180°/s (87 ± 15 Nm vs. 106 ± 7 Nm and 145 ± 13 Nm vs. 168 ± 14 Nm, respectively), with no significant differences in the H/Q ratio. Similarly, our study observed higher knee flexor and extensor strength across all angular velocities in professional athletes compared to junior athletes, while no differences were found in the H/Q ratios. These findings underscore the importance of using the H/Q ratio as a normative parameter, with deviations potentially indicating increased injury risk.

Regarding sex differences, our findings showed that female athletes, both in the professional and junior categories, had significantly lower knee flexion strength compared to their male counterparts, which may help explain the higher incidence of ACL injuries among women. However, no significant differences in H/Q ratio were observed between male and female athletes in the junior category. In the professional category, differences in H/Q ratios were found at 180°/s for both knees and at 30°/s for the left knee, with women reporting lower values in all three cases.

While continued assessment of the H/Q ratio is essential, particularly since values below 0.6 are considered a risk factor for ACL injury, in the case of female and junior athletes, a more comprehensive analysis is needed, including absolute knee flexor strength values. Rouis et al. [29] reported a knee flexor strength of 90 ± 15 N and extensor strength of 153 ± 25 N at 180°/s in female junior basketball players (Tier 3 [10]), which are lower than the values observed in our study (185 ± 51 N for flexor strength and 249 ± 69 N for extensor strength). However, it is important to note that our study included both male and female junior athletes, and the lever arm length was not specified, limiting the ability to draw definitive conclusions regarding absolute strength values.

In a study by Risberg et al. [30] involving professional soccer players (Tier 4 [10]), knee extension strength at 60°/s was reported as 146 ± 25 N, flexion strength as 85 ± 14 N, and the H/Q ratio as 0.59 ± 0.08. When comparing these values with those obtained in our study, we observed similar H/Q ratios at lower angular velocities (30°/s) but significantly higher absolute strength values in our sample. Specifically, professional athletes in our study demonstrated an extension strength of 486 ± 68 N and flexion strength of 255 ± 65 N, while junior athletes recorded 337 ± 83 N for extension and 184 ± 33 N for flexion. However, due to the absence of lever arm length data, no definitive conclusions can be drawn regarding absolute strength comparisons.

Baroni et al. [16], in their systematic review of professional soccer players (Tier 4 [10]), reported H/Q ratio values of 0.52 ± 0.08 at 30°/s, 0.65 ± 0.16 at 120°/s, and 0.67 ± 0.17 at 180°/s. These values are comparable to those observed in our study at 30°/s and 120°/s, but lower at 180°/s, where we found H/Q ratios of 0.77 ± 0.15 in professional athletes and 0.76 ± 0.16 in junior athletes.

In the study by Kabacinski et al. [31], college basketball players (Tier 3 [10]) reported H/Q ratios of 0.48 ± 0.07 in the right leg and 0.48 ± 0.04 in the left leg at 60°/s and 0.55 ± 0.05 in the right leg and 0.56 ± 0.03 in the left leg at 180°/s. These values are notably lower than those observed in the present study.

In the study by Brígido-Fernández et al. [21], a mean H/Q ratio of 0.54 ± 0.07 was reported at low angular velocity (60°/s) in female professional soccer players (Tier 4 [10]). In our study, at low velocity (30°/s), the mean H/Q ratio was similar across both professional and junior athletes. At 180°/s, Brígido-Fernández et al. [21] reported a mean H/Q ratio of 0.57 ± 0.09, which is relatively lower than the values observed in our study at the same angular velocity in both professional and junior athletes. It is important to note that their sample included only female professional soccer players, whereas our study involved both male and female professional and elite junior basketball players.

The systematic review by Baroni et al. [16] proposed a cut-off point of 0.6 for the H/Q ratio, below which the risk of knee joint injury increases. Based on this threshold, the values obtained in the present study at 30°/s would fall outside the safety range, suggesting a potential injury in both athlete categories. For hamstring muscle injuries, a cut-off point of 0.47 has been proposed; however, Dauty et al. [32] argue that this threshold should not be considered definitive, as only 2.7% of athletes with muscle injuries in their study had values below it. In contrast, Grygorowicz et al. [33] found that a conventional ratio cut-off of 0.66 offered greater sensitivity, and thus fewer false negatives, than the 0.47 threshold. However, using 0.47 as the cut-off provides greater specificity. These discrepancies highlight the difficulty in establishing a definitive H/Q ratio threshold for injury prediction. Consequently, it may be insufficient to rely solely on the H/Q ratio as a predictor of injury risk. Other variables, such as the absolute strength of the knee flexor–extensor complex, shown in our study to differ greatly between professional and junior athletes, may provide more meaningful insight, particularly given that H/Q ratios did not vary between groups.

Furthermore, with regard to muscular work, there is a limited number of studies on the subject [34], and, as with strength measurements, meaningful comparisons cannot be made due to lack of reported lever arm length in the existing literature.

The results of the present study indicate that there are no differences in BMI between male and female athletes in either the junior or professional basketball categories. These findings are consistent with those reported by Rosa-Guillamon et al. [34] in pre-adolescents and by Odobas et al. [35] in adult athletes. However, differences in BMI were observed between junior and professional players, with higher values recorded in the professional group.

Certain lower limb alterations have been associated with elevated BMI [36]. A variation in BMI implies differences in mass relative to height, which in turn requires the generation of different levels of force to achieve similar movement. In the present study, no significant differences in BMI were found between sexes. Therefore, it would be expected that comparable strength levels would be required to move a similar mass relative to height. However, this was not the case. This discrepancy may support the hypothesis that lower strength in the knee flexor-extensor musculature could contribute to an increased risk of ACL injuries.

## 5. Limitations

Another limitation of the present study is the sample size. However, it could not have been larger because it is a very specific and delimited population group.

It is important to determine the lever arm used for the measurements to correlate the data with other studies, which would facilitate consensus among the scientific community.

## 6. Conclusions

The data obtained in the present study show no differences between professional and elite junior athletes’ H/Q ratios (data used for ACL injury prevention). However, differences in quadriceps and hamstring musculature strength are observed in male and female basketball players.

Female athletes have lower strength data than males, and there are hardly any significant differences between sexes regarding H/Q ratios. Observing that there is a higher percentage of ACL injuries in the junior stage and female athletes, the H/Q ratio should be further assessed, since values below 0.6 represent a risk factor for ACL injury. In addition, in the case of women and young athletes, a more complete study with analysis of the absolute strength data of the knee flexors is required.

There are no differences in BMI between sexes, so to move the same mass with respect to height, they would need similar strength values, which does not occur.

## Figures and Tables

**Table 1:** Characterization of the subjects per sex and type of athlete (professional or junior) included in this study (mean ± standard deviation).

	Professional Athletes		
	Male Atheletes	Female Atheletes	*p*
*n*	27	16	
Mass (kg)	93.67 ± 9.90	72.25 ± 10.20	*p* ≤ 0.001 *
Height (m)	2.00 ± 0.09	1.79 ± 0.09	*p* ≤ 0.001 *
BMI	23.37 ± 1.19	22.4 ± 1.83	*p* = 0.059
	**Junior Athletes**		
	**Male Atheletes**	**Female Atheletes**	***p***
*n*	28	14	
Mass (kg)	66.00 ± 9.28	63.46 ± 9.93	*p* = 0.268
Height (m)	1.78 ± 0.06	1.70 ± 0.07	*p* ≤ 0.001 *
BMI	20.76 ± 2.46	22.34 ± 2.73	*p* = 0.081

The asterisk (*) indicates significant differences (*p* < 0.05).

**Table 2:** Overall characterization of the subjects per type of athlete (professional or junior) included in this study (mean ± standard deviation).

	Professional Athletes	Junior Atheletes	*p*
*n*	43	42	
Mass (kg)	85.70 ± 14.4	65.17 ± 9.40	*p* ≤ 0.001 *
Height (m)	1.92 ± 0.14	1.75 ± 0.07	*p* ≤ 0.001 *
BMI	23.01 ± 1.51	21.28 ± 2.63	*p* ≤ 0.001 *

The asterisk (*) indicates significant differences (*p* < 0.05).

**Table 3:** Mean, standard deviation, significance, and effect size of the differences between the dependent variables between athletes’ categories (professional and junior). Results are reported as Newtons (N) and Joules (J).

Variable ^1^	Angular Velocity	Knee	Professional Athletes Mean (±SD)	Junior Athletes Mean (±SD)	*p*-Value	Effect Size
Flexion peak strength (N)	30°/s	Right	255.14 ± 65.05 ^n^	184.26 ± 32.70 ^n^	<0.001 *	1.635
		Left	265.79 ± 70.42 ^n^	181.45 ± 32.35 ^n^	<0.001 *	1.763
	120°/s	Right	284.67 ± 76.33 ^n^	168.93 ± 44.09 ^n^	<0.001 *	1.961
		Left	283.58 ± 90.54 ^n^	170.88 ± 40.36 ^n^	<0.001 *	1.672
	180°/s	Right	307.58 ± 84.81 ^n^	185.35 ± 51.41 ^n^	<0.001 *	1.914
		Left	293.00 ± 88.92 ^nn^	187.55 ± 45.37 ^nn^	<0.001 *	1.610
Extension peak strength (N)	30°/s	Right	485.70 ± 67.86 ^n^	336.86 ± 82.67 ^nn^	<0.001 *	2.069
		Left	466.51 ± 72.47 ^n^	341.79 ± 73.24 ^n^	<0.001 *	1.846
	120°/s	Right	415.84 ± 83.74 ^n^	236.98 ± 75.90 ^n^	<0.001 *	2.470
		Left	411.95 ± 82.46 ^n^	253.07 ± 70.94 ^n^	<0.001 *	2.391
	180°/s	Right	399.90 ± 83.65 ^n^	249.35 ± 68.65 ^n^	<0.001 *	2.203
		Left	396.03 ± 87.30 ^n^	248.48 ± 59.38 ^nn^	<0.001 *	2.142
Knee work (J)	30°/s	Right	691.53 ± 172.21 ^n^	450.14 ± 87.32 ^n^	<0.001 *	2.053
		Left	685.84 ± 181.74 ^n^	455.95 ± 87.44 ^nn^	<0.001 *	1.891
	120°/s	Right	1001.91 ± 267.82 ^n^	517.02 ± 153.61 ^n^	<0.001 *	2.526
		Left	1026.53 ± 322.70 ^nn^	568.98 ± 144.02 ^n^	<0.001 *	2.118
	180°/s	Right	1843.87 ± 523.50 ^n^	1012.19 ± 310.31 ^n^	<0.001 *	2.128
		Left	1828.42 ± 487.81 ^n^	1023.35 ± 262.29 ^n^	<0.001 *	2.244
H/Q ratio	30°/s	Right	0.53 ± 0.11 ^n^	0.57 ± 0.11 ^n^	0.145	−0.329
		Left	0.57 ± 0.11 ^n^	0.54 ± 0.09 ^n^	0.273	0.238
	120°/s	Right	0.69 ± 0.15 ^n^	0.76 ± 0.24 ^n^	0.338	−0.451
		Left	0.69 ± 0.18 ^n^	0.70 ± 0.17 ^nn^	0.972	−0.250
	180°/s	Right	0.77 ± 0.15 ^nn^	0.76 ± 0.16 ^n^	0.849	−0.083
		Left	0.74 ± 0.16 ^n^	0.77 ± 0.15 ^nn^	0.459	−0.354

SD—standard deviation; ^1^ strength peak is the maximum value of the repetitions performed; * values reporting significant differences; ^n^ the variable is normally distributed; ^nn^ the variable is non-normally distributed.

**Table 4:** Mean, standard deviation, significance, and effect size of the differences between the dependent variables between sexes in professional athletes. Results are reported as Newtons (N) and Joules (J).

Variable ^1^	Angular Velocity	Knee	Male Athletes Mean (±SD)	Female Athletes Mean (±SD)	*p*-Value	Effect Size
Flexion peak strength (N)	30°/s	Right	292.06 ± 59.59	225.00 ± 49.88	0.002 *	1.22
		Left	290.44 ± 59.40	234.75 ± 41.43	0.005 *	1.09
	120°/s	Right	321.00 ± 77.46	243.44 ± 52.50	0.005 *	1.17
		Left	327.69 ± 91.36	234.19 ± 53.01	<0.001 *	1.25
	180°/s	Right	362.36 ± 78.61	246.77 ± 48.49	<0.001 *	1.75
		Left	347.36 ± 90.20	238.62 ± 44.77	0.002 *	1.51
Extension peak strength (N)	30°/s	Right	510.94 ± 60.27	448.87 ± 63.60	0.011 *	1
		Left	488.69 ± 81.27	445.88 ± 48.60	0.051	0.64
	120°/s	Right	439.50 ± 91.38	375.94 ± 70.70	0.051	0.78
		Left	448.25 ± 86.18	375.56 ± 59.10	0.007 *	0.98
	180°/s	Right	429.93 ± 91.42	355.85 ± 60.22	0.017 *	0.95
		Left	424.50 ± 93.66	352.85 ± 65.94	0.019 *	0.88
Knee work (J)	30°/s	Right	790.50 ± 146.70	570.63 ± 101.70	<0.001 *	1.74
		Left	784.88 ± 164.93	567.69 ± 101.14	<0.001 *	1.59
	120°/s	Right	1129.19 ± 234.91	805.69 ± 156.78	<0.001 *	1.62
		Left	1134.88 ± 221.34	812.50 ± 141.51	<0.001 *	1.73
	180°/s	Right	2108.64 ± 489.27	1429.08 ± 292.14	<0.001 *	1.67
		Left	2070.36 ± 447.03	1463.38 ± 275.26	0.001 *	1.62
H/Q ratio	30°/s	Right	0.57 ± 0.11	0.51 ± 0.12	0.086	0.61
		Left	0.60 ± 0.10	0.53 ± 0.09	0.023 *	0.72
	120°/s	Right	0.74 ± 0.15	0.66 ± 0.15	0.08	0.51
		Left	0.74 ± 0.18	0.63 ± 0.17	0.051	0.61
	180°/s	Right	0.85 ± 0.10	0.71 ± 0.17	0.048 *	1
		Left	0.82 ± 0.15	0.69 ± 0.16	0.038 *	0.83

SD—standard deviation; ^1^ strength peak is the maximum value of the repetitions performed; * values reporting significant differences.

**Table 5:** Mean, standard deviation, significance, and effect size of the differences between the dependent variables between sexes in junior athletes. Results are reported as Newtons (N) and Joules (J).

Variable ^1^	Angular Velocity	Knee	Male Athletes Mean (±SD)	Female Athletes Mean (±SD)	*p*-Value	EFFECT SIZE
Flexion peak strength (N)	30°/s	Right	191.93 ± 41.08	170.29 ± 24.81	0.21	0.64
		Left	191.00 ± 37.98	166.57 ± 24.14	0.056	0.77
	120°/s	Right	176.79 ± 50.84	150.86 ± 28.28	0.104	0.63
		Left	178.43 ± 46.56	149.71 ± 26.85	0.039 *	0.77
	180°/s	Right	185.00 ± 61.33	170.5 ± 40.44	0.571	0.28
		Left	196.57 ± 48.73	165.54 ± 27.54	0.061	0.77
Extension peak strength (N)	30°/s	Right	367.14 ± 78.58	287.5 ± 76.86	0.021 *	1.02
		Left	358.50 ± 61.91	310.5 ± 81.13	0.077	0.66
	120°/s	Right	272.50 ± 78.78	190.93 ± 63.21	0.006 *	1.14
		Left	276.93 ± 67.02	207.64 ± 60.57	0.009 *	1.08
	180°/s	Right	255.86 ± 78.18	206.64 ± 62.69	0.069	0.69
		Left	255.50 ± 69	200.46 ± 52.69	0.054	0.89
Knee work (J)	30°/s	Right	469.43 ± 86.69	404.07 ± 71.85	0.031 *	0.82
		Left	482.64 ± 77.98	407.29 ± 78.90	0.021 *	0.96
	120°/s	Right	570.29 ± 147.53	435.36 ± 144.86	0.024 *	0.92
		Left	610.36 ± 117.57	474.64 ± 113.74	0.006 *	1.17
	180°/s	Right	1067.64 ± 327.50	773.43 ± 268.75	0.019 *	0.98
		Left	1045.07 ± 303.77	815.23 ± 209.88	0.033 *	0.87
H/Q ratio	30°/s	Right	0.53 ± 0.09	0.62 ± 0.14	0.094	−0.77
		Left	0.54 ± 0.08	0.56 ± 0.12	0.701	−0.24
	120°/s	Right	0.67 ± 0.17	0.86 ± 0.29	0.069	−0.79
		Left	0.65 ± 0.10	0.76 ± 0.22	0.482	−0.65
	180°/s	Right	0.74 ± 0.15	0.85 ± 0.15	0.062	−0.74
		Left	0.80 ± 0.22	0.86 ± 0.16	0.402	−0.28

SD—standard deviation; ^1^ strength peak is the maximum value of the repetitions performed; * values reporting significant differences.

## Data Availability

The original contributions presented in the study are included in the article; further inquiries can be directed to the corresponding author.
